# Upregulated HSP27 in human breast cancer cells reduces Herceptin susceptibility by increasing Her2 protein stability

**DOI:** 10.1186/1471-2407-8-286

**Published:** 2008-10-04

**Authors:** Se Hun Kang, Keon Wook Kang, Kyung-Hee Kim, Bumi Kwon, Seok-Ki Kim, Ho-Young Lee, Sun-Young Kong, Eun Sook Lee, Sang-Geun Jang, Byong Chul Yoo

**Affiliations:** 1 Research Institute and Hospital, National Cancer Center, Republic of Korea; 2 Department of Nuclear Medicine, School of Medicine, Seoul National University, Seoul, Korea

## Abstract

**Background:**

Elucidating the molecular mechanisms by which tumors become resistant to Herceptin is critical for the treatment of Her2-overexpressed metastatic breast cancer.

**Methods:**

To further understand Herceptin resistance mechanisms at the molecular level, we used comparative proteome approaches to analyze two human breast cancer cell lines; Her2-positive SK-BR-3 cells and its Herceptin-resistant SK-BR-3 (SK-BR-3 HR) cells.

**Results:**

Heat-shock protein 27 (HSP27) expression was shown to be upregulated in SK-BR-3 HR cells. Suppression of HSP27 by specific siRNA transfection increased the susceptibility of SK-BR-3 HR cells to Herceptin. In the presence of Herceptin, Her2 was downregulated in both cell lines. However, Her2 expression was reduced by a greater amount in SK-BR-3 parent cells than in SK-BR-3 HR cells. Interestingly, co-immunoprecipitation analysis showed that HSP27 can bind to Her2. In the absence of Herceptin, HSP27 expression is suppressed and Her2 expression is reduced, indicating that downregulation of Her2 by Herceptin can be obstructed by the formation of a Her2-HSP27 complex.

**Conclusion:**

Our present study demonstrates that upregulated HSP27 in human breast cancer cells can reduce Herceptin susceptibility by increasing Her2 protein stability.

## Background

The epidermal growth factor receptor (EGFR)-related tyrosine kinase Her2 is an important therapeutic target in breast cancer. Herceptin is active against Her2-overexpressing metastatic breast cancers. However, the objective response rates to Herceptin monotherapy are low, with median 9-month response rates ranging from 12–34% [1]. At present, Herceptin is administered in combination with chemotherapies involving paclitaxel or docetaxel, which increase response rates, time to disease progression, and overall survival compared with Herceptin monotherapy. However, most patients who achieve an initial response to Herceptin-based regimens generally acquire resistance within 1 year [2-6].

Determining the molecular mechanisms by which tumors become resistant to Herceptin-mediated cytotoxicity is critical to improving the survival of metastatic breast cancer patients with tumors that overexpress Her2. Numerous efforts have been made to identify the mechanisms underlying Herceptin resistance [see reviews [7] and [8]]. For example, Her2 mutations or low Her2 expression levels reduced the interaction between Her2 and Herceptin, and led to increased compensatory signaling from other Her receptors [9-11]. However, our understanding of Herceptin resistance remains limited.

In the present study we sought to further our understanding of Herceptin resistance mechanisms at the molecular level. The biological impact of overexpressed heat-shock protein 27 (HSP27) in the human breast cancer cell line SK-BR-3 with Herceptin resistance (SK-BR-3 HR) is discussed.

## Methods

### Human breast cancer cell lines

The human breast cancer cell lines SK-BR-3, AU565, HCC1569, HCC70, and MCF7 were obtained from the American Type Culture Collection (ATCC, Manassas, VA). The Herceptin-resistant human breast cancer cell line JIMT-1 was purchased from the German Collection of Microorganisms and Cell Cultures (Deutsche Sammlung von Mikroorganismen und Zellkulturen GmbH, Braunschweig, Germany). SK-BR-3 HR cells, which are resistant to Herceptin (Genentech, Inc., South San Francisco, CA), were spontaneously derived from SK-BR-3 cells by increasing the generation-to-generation passage.

### Genotyping of SK-BR-3 and its Herceptin-resistant-derivative cell line, SK-BR-3 HR

The total amount of genomic DNA was extracted using the TRI reagent according to the manufacturer's instructions (Molecular Research Center, Cincinnati, OH, USA). PCR amplification was performed using the AmpFlSTR Profiler™ PCR Amplification Kit (Applied Biosystems, Oster City, CA) to amplify the D3S1358, vWA, FGA, amelogenin, TH01, TPOX, CSF1PO, D5S818, D13S317, and D7S820 loci in sample genomic DNA. PCR assays were performed according to the manufacturer's recommendations except that the PCR volume was 50 μL and contained 2 ng of template DNA. The PCR conditions were 95°C for 11 minutes, followed by 28 cycles at 94°C for 1 minute, 59°C for 1 minute, and 72°C for 1 minute. A final extension was conducted at 60°C for 45 minutes, and the products were then maintained at 25°C. Separation and detection of the amplified product was performed using an ABI 3730 Genetic Analyser (Applied Biosystems) with GeneScan 2.1 and Genotyper 2.0 software.

### 3- [4,5-Dimethylthiazol-2-yl]-2,5-diphenyltetrazolium bromide (MTT) assay

A colorimetric assay using the tetrazolium salt MTT was used to assess cell proliferation after HSP27 suppression. MTT assays were performed as described in a previous report [12]. Briefly, 0.18 mL cultures containing equal numbers of cells were incubated in each well, to which 0.02 mL of 10 × Herceptin (Genentech, Inc.) or phosphate-buffered saline (PBS, an untreated 100% survival control) was added. After 4 days of culture, 0.1 mg of MTT was added to each well, and incubated at 37°C for a further 4 hours. Plates were centrifuged at 450 × g for 5 minutes at room temperature and the supernatant was removed. Dimethyl sulfoxide (0.15 mL) was added to each well to solubilize the crystals, and the plates were immediately read at 540 nm using a scanning multiwell spectrometer (Bio-Tek instruments Inc., Burlington, VT). All experiments were performed three times, and the IC_50 _(μg/mL) values are presented as means ± standard deviation.

### Western-blot analysis

Western-blot analysis was performed as described previously [12]. Briefly, cell homogenates containing equivalent amounts of protein were centrifuged at 4,000 × g, and the supernatant fractions were separated by SDS-PAGE. Following electrophoresis, the proteins were transferred to polyvinylidene fluoride (PVDF) membranes (Millipore, Bedford, USA) blocked by incubation for 2 hours at 4°C in 1% Tween 20-TBS buffer containing 1.5% non-fat dry milk (Bio-Rad, Richmond, USA) and 1 mM of MgCl_2_. Membranes were incubated for 2 hours at room temperature with primary antibodies against Her2 (Dako, Glostrup, Denmark), HSP27 (Abcam, Cambridgeshire, UK), fibrillarin (Abcam), or actin (Sigma, St Louis, MO). Membranes were washed three times with blocking solution, for 15 minutes at a time, and incubated with diluted horseradish-peroxidase (HRP)-conjugated secondary antibody (Southern Biotech, Birmingham, AL) for 1 hour at room temperature. This was followed by washing with blocking solution (3 × 15 minutes), incubation with WEST-ZOL^® ^plus chemiluminescence reagent (iNtRON Biotechnology, Gyeonggi, Korea) for 1 minute, and exposure to film (Kodak Blue XB-1, Rochester, NY).

### 2-DE

Two-dimensional electrophoresis (2-DE) analysis was performed as previously described [12]. Briefly, 0.15 mg protein samples were applied to 13 cm immobilized pH 3–10 non-linear gradient strips (Pharmacia Biotechnology, Uppsala, Sweden). Proteins were focused at 8,000 V within 3 hours. The second-dimension separation involved 12% polyacrylamide gels (chemicals from Serva, Heidelberg, Germany and Bio-Rad, Hercules, CA). 2-DE gels were stained with Colloidal Coomassie Blue (Invitrogen, Carlsbad, CA) for 24 hours and were then destained with deionized water.

### Matrix-assisted laser desorption ionization-mass spectrometry (MALDI-MS) and database searching

Sections of 2-DE gels containing proteins of interest were excised, destained with 50% acetonitrile in 0.1 M ammonium bicarbonate, and dried in a SpeedVac evaporator. Dried gel pieces were reswollen with 30 μL of 25 mM sodium bicarbonate at pH 8.8, containing 50 ng of trypsin (Promega, Madison, WI) at 37°C overnight. α-Cyano 4 hydroxycinnamic acid (20 mg) (Bruker Daltonics, Bremen, Germany) was dissolved in 1 mL acetone:ethanol (1:2, v/v), and 0.5 μL of the matrix solution was mixed with an equivalent volume of the sample. Analysis was performed using an Ultraflex TOF/TOF system (Bruker Daltonics).

The Ultraflex TOF/TOF system was operated in the positive-ion-reflect mode. Each spectrum was the average of 250–450 laser shots. Mass spectra were first calibrated in the closed external mode using the peptide calibration standard II (Bruker Daltonics), and sometimes also in the internal statistical mode to achieve maximum calibration mass accuracy, and then analyzed with FlexAnalysis software, version 2.4 (Bruker Daltonics). Peptide mass peaks from each spectrum were analyzed by the Mascot peptide mass fingerprinting search tool  using BioTools software, version 3.0 (Bruker Daltonics).

The search included peaks with a signal-to-noise (S/N) ratio greater than 3. The peak list for each sample was compared with the non-redundant Mass Spectrometry Protein Sequence Database (MSDB) for protein identification. Standard settings included the following: enzyme, trypsin; missed cleavage, one; fixed modifications, none selected; variable modifications, oxidized methionine; protein mass, blank; mass values, MH+ (mono-isotopic); mass tolerance, between 75 and 100 ppm.

### Subcellular fractionation

Nuclear fractions of cells were isolated from cytoplasm using the nuclei isolation kit 'Nuclei PURE Prep' (Sigma-Aldrich, St Louis, MO), as recommended by the manufacturer. Membrane fractionation was performed as described previously with the following modifications [13]. Briefly, cells were vortexed vigorously for 5 min in cold lysis buffer containing 40 mM of Tris and 150 U/mL of benzonase, and then homogenized on ice. Vortexing and homogenization were repeated. The cell homogenate was centrifuged at 12,000 × *g *for 10 min at room temperature. The insoluble pellet was washed twice with lysis buffer and resuspended in membrane fractionation buffer containing 5 M urea, 2 M thiourea, 2% w/v CHAPS, 40 mM Tris and 100 mM DTT. The vortexing, homogenization and centrifugation process was then repeated. The supernatant was collected as the membrane fraction.

### Immunoprecipitation

All procedures were performed at 4°C unless otherwise specified. Approximately 10^7 ^cells in 1 ml of cold 1 × RIPA buffer containing protease inhibitors (Roche Diagnostics) were incubated on ice for 30 minutes with occasional mixing. Cell lysates were centrifuged at 12,000 × g for 10 minutes, and the supernatants were collected without disturbing the pellet. The supernatants were mixed with primary antibodies against Her2 (Dako) and HSP27 (Abcam), and incubated for 2 hours on a rocking platform. Prepared protein G sepharose beads (100 μL) (GE Health Care Life Sciences) were added and were further incubated on ice for 1 hour on a rocking platform. The mixture was centrifuged at 10,000 × g for 30 seconds, and the supernatant was then completely removed. Protein G sepharose beads were washed five times with 1 mL of cold 1 × RIPA to minimize the background. Next, 100 μL of 2 × SDS sample buffer was added to the bead pellet, and the mixture was heated at 100°C for 10 minutes. After boiling, immunoprecipitates were centrifuged at 10,000 × g for 5 minutes, and the supernatants were collected for Western-blot analyses.

### siRNA synthesis and transfection

Validated siRNA (Cat No. SI00300496) against HSP27 and non-silencing (NS) control siRNA (5'-AATTCTCCGAACGTGTCACGT-3') were obtained from Qiagen, Chatsworth, CA. Transfection with siRNAs was performed using HiferFect transfection reagent (Qiagen), according to the manufacturer's instructions. Briefly, 2 μL of 20 μM siRNA solution and 20 mL of the transfection reagent were incubated in 100 mL of serum-free RPMI 1,640 medium for 10 minutes to facilitate complex formation. The resulting mixture (final concentration of 5 nM) was added to the Herceptin-resistant SK-BR-3 (4 × 10^5^), and incubated in a 60 mm tissue culture dish with 4 mL RPMI 1640.

## Results

### Establishment of SK-BR-3 HR, a human breast cancer cell line with Herceptin resistance

**Figure 1:**
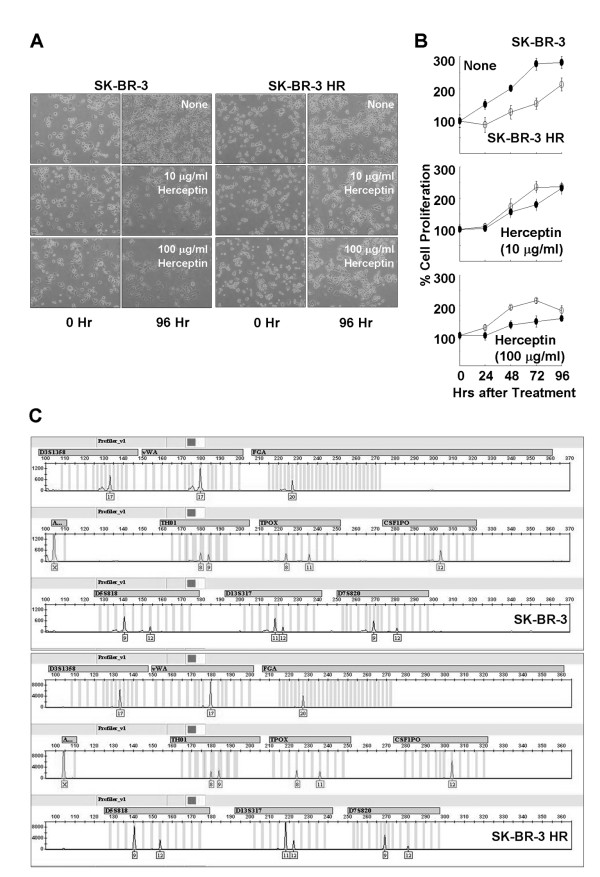
**Establishment of human breast cancer cell line SK-BR-3 with Herceptin resistance**. (a) Microscopy images showing increased cell proliferation of the SK-BR-3 derivative cell line SK-BR-3 HR in the presence of Herceptin. (b) Differential proliferation rate of SK-BR-3 HR in the presence of Herceptin. Data are means ± standard deviations of three independent experiments. (c) Genotyping of SK-BR-3 and SK-BR-3 HR, showing the two cell lines have the same genetic identity.

The Human breast cancer cell line SK-BR-3 was cultured according to the ATCC instructions, and the derivative cell line from SK-BR-3 with reduced Herceptin susceptibility was generated spontaneously by increasing generation-to-generation passage. Microscopy images in Fig. 1a show increased proliferation of the Herceptin-resistant SK-BR-3 derivative cell line (SK-BR-3 HR) in the presence of Herceptin. Increased proliferation of SK-BR-3 HR cells in the presence of Herceptin was confirmed by the MTT time-course assay (Fig. 1b). However, these two cell lines, parent SK-BR-3 and the Herceptin-resistant SK-BR-3 HR, are genetically identical (Fig. 1c).

### Identification of overexpressed HSP27 in SK-BR-3 HR

**Figure 2:**
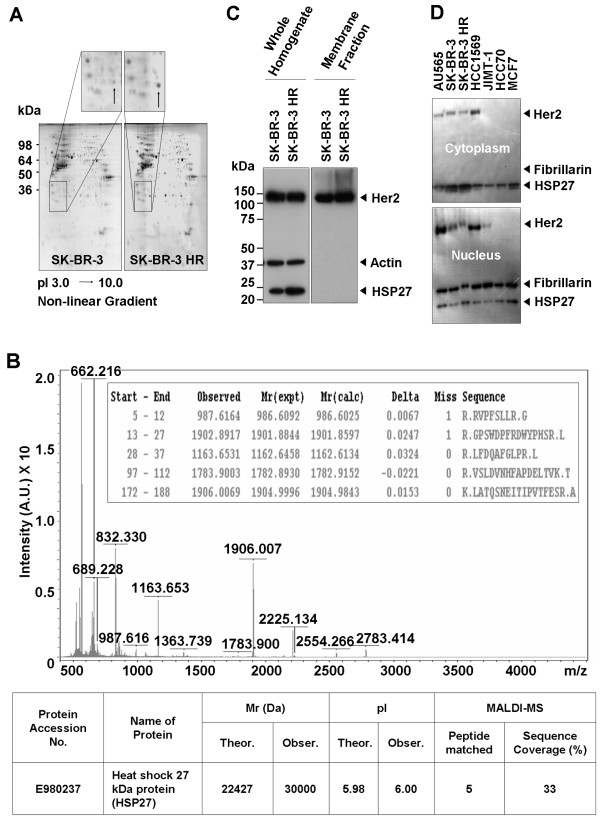
**Identification of HSP27 overexpressed in SK-BR-3 HR**. (a) Typical pattern of two-dimensional (2-DE) gel electrophoresis analysis of SK-BR-3 and SK-BR-3 HR cells. (b) Identification of HSP27 by MALDI-MS analysis. The protein spot indicated in the enlarged image in Figure 2A was in-gel-digested by trypsin and subjected to MALDI-MS analysis. The protein was identified as human HSP27. (c) Western-blot analysis to confirm overexpressed HSP27 in SK-BR-3 HR cells. (d) Subcellular levels of HSP27 and Her2 in seven individual human breast cancer cell lines.

To verify the differential protein expression between the SK-BR-3 and SK-BR-3 HR cell lines, total proteins were isolated from SK-BR-3 and SK-BR-3 HR cells and subjected to 2-DE. A strongly stained protein spot from SK-BR-3 HR cells (enlarged partial 2-DE gels on Fig. 2a) was in-gel digested for MALDI-MS analysis, and shown to be human HSP27 (Fig. 2b). Increased levels of HSP27 in SK-BR-3 HR cells were confirmed by Western-blot analysis (Fig. 2c). However, there were no differences in Her2 expression between the two cell lines (Fig. 2c, d). The subcellular levels of HSP27 and Her2 were investigated in seven human breast cancer cell lines, including SK-BR-3 and SK-BR-3 HR (Fig. 2d). Most HSP27 was localized in the cytoplasm of the breast cancer cell lines tested. However, there was no correlation between Herceptin susceptibility in breast cancer cell lines and HSP27 expression (Fig. 2d). HSP27 expression in JIMT-1, which is a cell line that has previously been reported to be Herceptin resistant, was lower than in SK-BR-3 HR (Fig. 2d).

### Increased susceptibility of SK-BR-3 HR to Herceptin after suppression of HSP27

**Figure 3:**
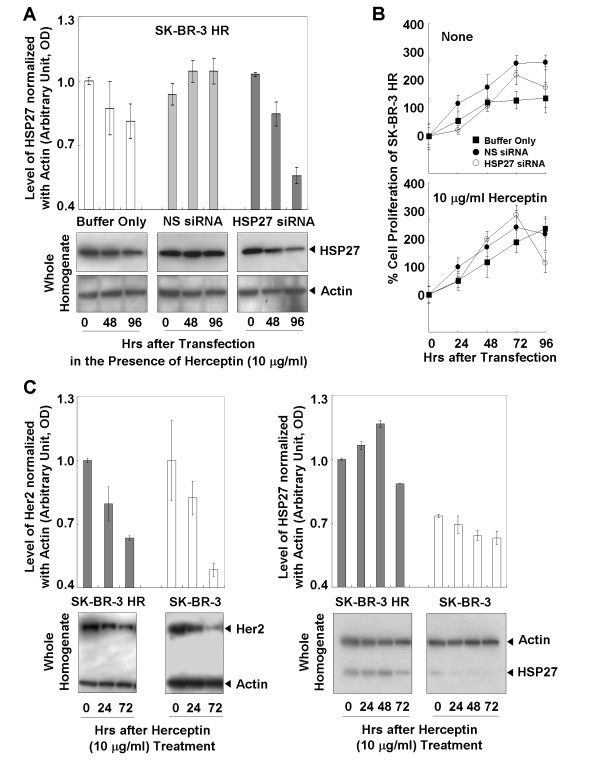
**Effects of suppressed HSP27 on the susceptibility of SK-BR-3 HR to Herceptin**. (a) Suppressed HSP27 after transfection with siRNAs in the presence of Herceptin. (b) MTT analysis showed increased Herceptin susceptibility of SK-BR-3 HR after HSP27 suppression. (c) Effect of Herceptin on the levels of Her2 and HSP27. Altered total levels of HSP27 and Her2 by Herceptin treatment. Treatment of Herceptin led to reductions in HSP27 and Her2 levels in the homogenates of SK-BR-3 and SK-BR-3 HR cells. Data (a, b, c) are means ± standard deviations of three independent experiments.

SK-BR-3 HR was transfected with siRNA against HSP27 (Fig. 3a), and the proliferation of cells was assessed (Fig. 3b). At 96 hours after transfection in the presence of Herceptin, the level of HSP27 expression was decreased to about 55% the original level, and the rate of cell proliferation was about 65% of that for controls transfected with buffer only or non-silencing (NS) siRNAs (Fig, 3b, lower panel).

### Effect of Herceptin on the levels of HSP27 and Her2 protein

Compared with the SK-BR-3 HR cells, there were significant reductions in Her2 expression in SK-BR-3 parent cells after treatment with Herceptin (Fig. 3c, left panel). The levels of HSP27 were essentially not changed by the Herceptin treatment (Fig. 3c, right panel).

### Interaction of HSP27 with Her2 and its effect on Her2 stability

**Figure 4:**
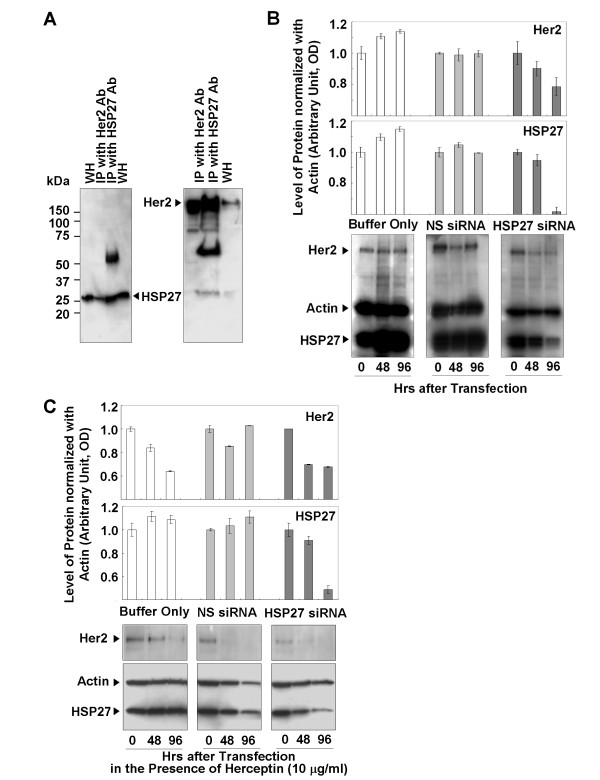
**Interactions between HSP27 and Her2, and reduced Her2 levels after HSP27 suppression**. (a) Western-blot analysis of immunoprecipitates of anti-Her2 and anti-HSP2 antibodies. (b) Reduced Her2 levels associated with HSP27 suppression. (c) Reduced levels of Her2 in control and HSP27-suppressed cells in the presence of Herceptin. Data (b, c) are means ± standard deviations of three independent experiments.

To test the interaction between HSP27 and Her2, immunoprecipitate of the anti-Her2 antibody was proved by the anti-HSP27 antibody, and vice versa (Fig. 4a). Immunoreactive signals of Her2 and HSP27 were clearly detected in the immunoprecipitates of anti-HSP27 and anti-Her2, respectively (Fig. 4a). Without treatment of Herceptin, the HSP27 in SK-BR-3 HR was suppressed, and the level of Her2 was compared to that of the controls transfected by either buffer only or NS siRNA (Fig. 4b). In the absence of Herceptin, the Her2 protein level was not affected by siRNA transfection conditions, but HSP27 suppression was accompanied by a decrease in the Her2 protein level (Fig. 4b). However, in the presence of Herceptin Her2 protein was decreased not only in cells transfected by HSP27 siRNA but also in the controls (Fig. 4c).

## Discussion and conclusion

In the present study, to understand Herceptin-resistance mechanisms in human breast cancer cells, the Herceptin-resistant human breast cancer cell line SK-BR-3 HR was generated from the parent cell line SK-BR-3 (Fig. 1). Comparative proteomic analyses of these two cell lines revealed that HSP27 expression was upregulated in SK-BR-3 HR cells (Fig. 2). HSPs are known to play important roles in folding, intracellular localization, and degradation of cellular proteins, but the cellular role of HSP27 in cancers is not yet completely understood. Recently, there have been reports that high HSP27 expression levels are associated with a poor prognosis for specific cancers including gastric, liver, and prostate carcinomas, and osteosarcomas [see the review, [14]]. Furthermore, expression of HSP27 in primary breast cancers is associated with a short survival for node-negative patients, and increased HSP27 expression levels have been found in highly metastatic variant breast cancer cells [15,16]. However, osteolytic bone metastases of human breast cancer cells are reduced by HSP27 overexpression [17].

In addition to being a clinicopathological factor, upregulated HSP27 expression has also been proposed to be predictive of poor response to some anticancer treatments for breast cancer, non-small cell lung cancer, and bladder cancer [14,18-20]. For example, HSP27 overexpression inhibits doxorubicin-induced apoptosis in human breast cancer cells [21]. However, increased levels of HSP27 expression were not significantly associated with response to tamoxifen, time to treatment failure, or survival in an estrogen-receptor-positive breast cancer population [22].

HSP27 is highly expressed in Her2-positive tumors [23]. HSP27 is one of the downstream effectors of p38 MAP kinase-mediated matrix metalloproteinase type 2 activation and is a modulator of Stat3-regulated apoptosis [24,25]. Ser78 phosphorylation of HSP27 is mainly regulated by the Her2-p38 MAPK pathway, and is significantly correlated with Her2- and lymph-node positivity [23]. Phosphorylation of HSP27 may play a role in the response of breast cancer cells to the anticancer drug [26]. However a detailed understanding of the role of HSP27 in Herceptin-resistance mechanisms has yet to be achieved. Our present results provide further evidence to support the involvement of HSP27 in Herceptin resistance in human breast cancer cells (Fig. 3). Changes in the proliferation rate of SK-BR-3 cells with different HSP27 levels may provide a clue. Previous studies have reported reduced proliferation levels of the human breast cancer cell line MDA-MB-231 and the human laryngeal cancer cell line Hep-2 after transfection with an HSP27-overexpressing vector [27,28]. In agreement with these results, we found a slow proliferation rate of SK-BR-3 HR with upregulated HSP27 (Fig. 1b, upper panel). A decreased proliferation rate was observed after HSP27 suppression in the presence of Herceptin, but HSP27 suppression alone did not affect the rate of cell proliferation (Fig. 3b). A slow proliferation rate of cancer cells is known to be a mechanism to minimize the effects of anticancer agents, so overexpression of HSP27 may reduce the Herceptin susceptibility of human breast cancer cells by affecting the proliferation rate [29-31].

Her2 expression was shown to be downregulated in the presence of Herceptin in two different human breast cancer cell lines, SK-BR-3 (Fig. 3c) and MDA453 [32]. Degradation of the Her2 protein has been proposed as a mechanism by which Herceptin acts on Her2-positive breast cancer cells [8]. Our present results are an interesting example of how Her2 levels are affected by Herceptin (Fig. 3c, left panel). In the presence of Herceptin, there were greater reductions in the levels of Her2 in SK-BR-3 cells compared with SK-BR-3 HR cells (Fig. 3c, left panel), even though there was no mutation in the Her2 gene in either cell line (data not shown). Our results also indicate that Herceptin-induced reductions in Her2 levels can be minimized in SK-BR-3 HR cells. Immunoprecipitation analysis of the interaction between Her2 and HSP27 demonstrated the binding capacity of HSP27 to Her2 (Fig. 4a). Protein-protein binding can often increase protein stability and upregulated HSP27 expression levels have been reported in Her2-positive breast tumors [33]. Therefore, we hypothesized that HSP27 may stabilize Her2. Interestingly, suppression of HSP27 was associated with reductions in Her2 levels in the absence of Herceptin (Fig. 4b). It is still not clear whether HSP27 can interact with Her2 at different subcellular locations. However, both molecules were detected not only in the cytoplasm but also in the nucleus (Fig. 2d), suggesting that Her2 may be stabilized by interaction with HSP27 regardless of subcellular localization. Overall, our findings indicate that upregulation of HSP27 can make SK-BR-3 HR cells less sensitive to Herceptin by minimizing the Herceptin-induced Her2 degradation.

In conclusion, our findings demonstrate that upregulation of HSP27 in human breast cancer cells can reduce Herceptin susceptibility by increasing Her2 protein stability. The development of specific agents to block HSP27 may overcome Herceptin resistance and improve the survival of breast cancer patients [34].

## Competing interests

The authors declare that they have no competing interests.

## Authors' contributions

SHK, K–HK, BK, S–KK, H–YL, S–YK and S–GJ participated in data acquisition. ESL supervised and with KWK and BCY participated in the design and coordination of the study. BCY drafted and wrote the manuscript, is the guarantor. All authors read and approved the final manuscript

## Pre-publication history

The pre-publication history for this paper can be accessed here:


